# Supplementary material for: Seminoma and Embryonal Carcinoma Footprints Identified by Analysis of Integrated Genome-Wide Epigenetic and Expression Profiles of Germ Cell Cancer Cell Lines
Source: PLoS One. 2014 Jun 2;9(6):e98330. doi: 10.1371/journal.pone.0098330 (PMC4041891; doi:10.1371/journal.pone.0098330)
Supplement: File S1 — ZIP file containing DMRforPairs output for significant regions. Please start from the html files. (ZIP) [file pone.0098330.s008.zip › figures/9729.pdf]

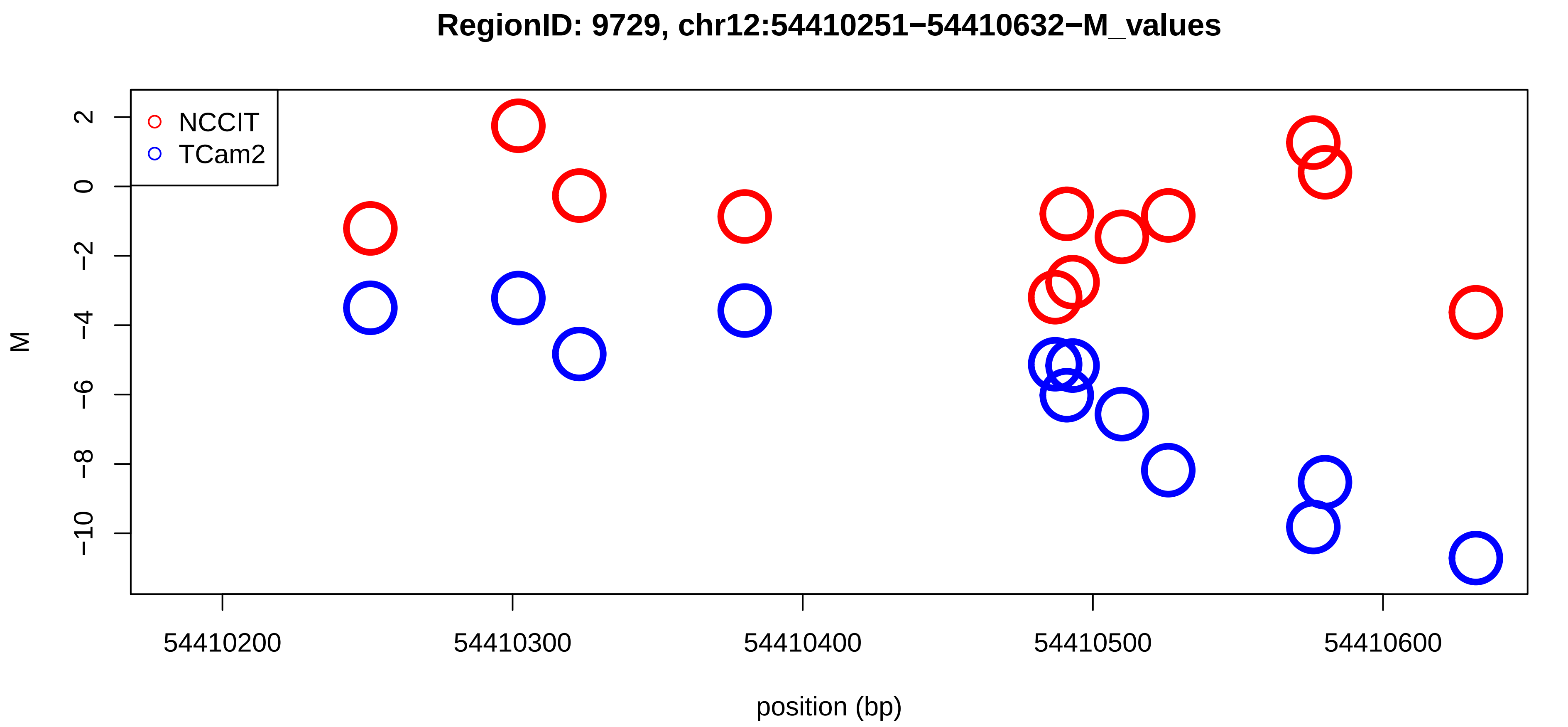

RegionID: 9729, chr12:54410251−54410632−M_values
NCCIT
TCam2
M
position (bp)
54410200
54410300
54410400
54410500
54410600
2
0
−2
−4
−6
−8
−10

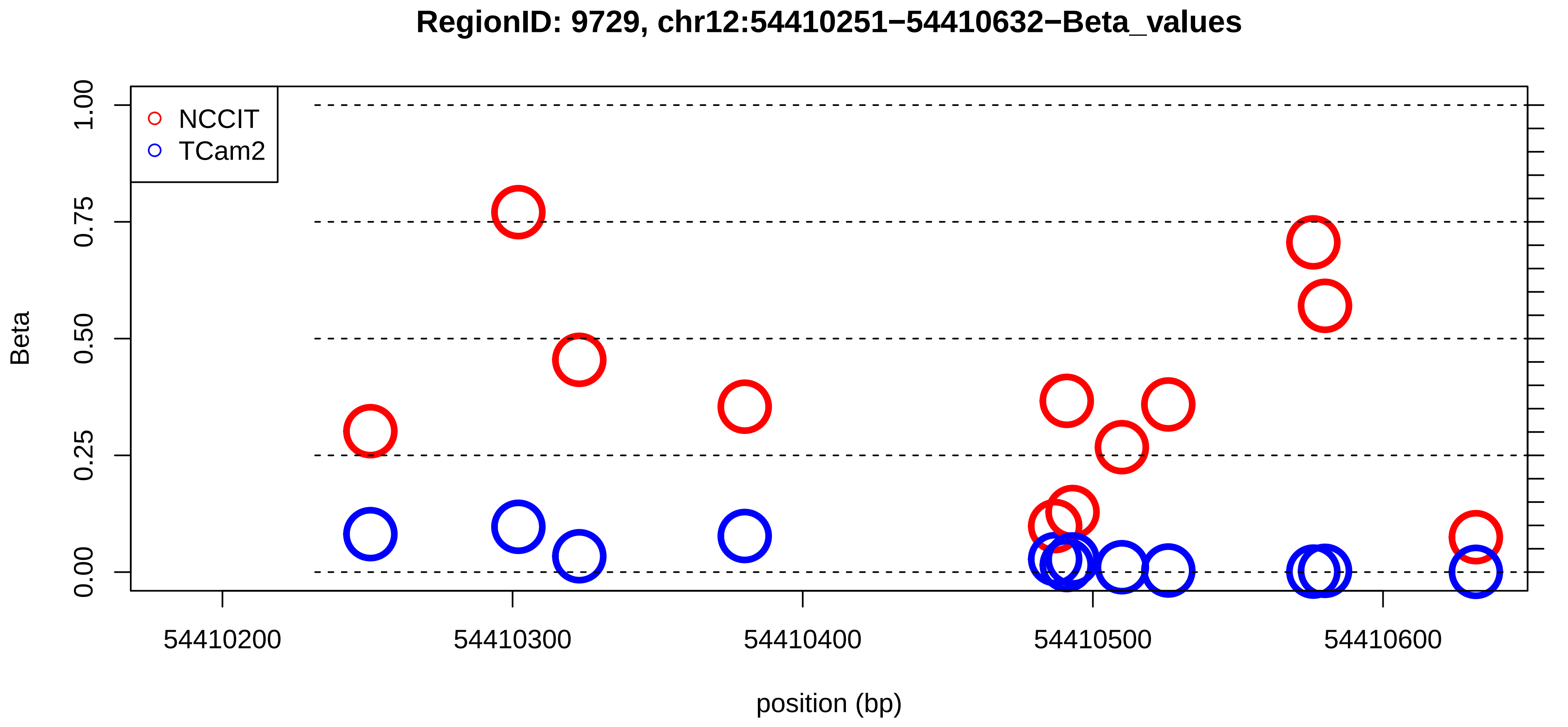

RegionID: 9729, chr12:54410251−54410632−Beta_values
NCCIT
TCam2
Beta
position (bp)
54410200
54410300
54410400
54410500
54410600
1.00
0.75
0.50
0.25
0.00

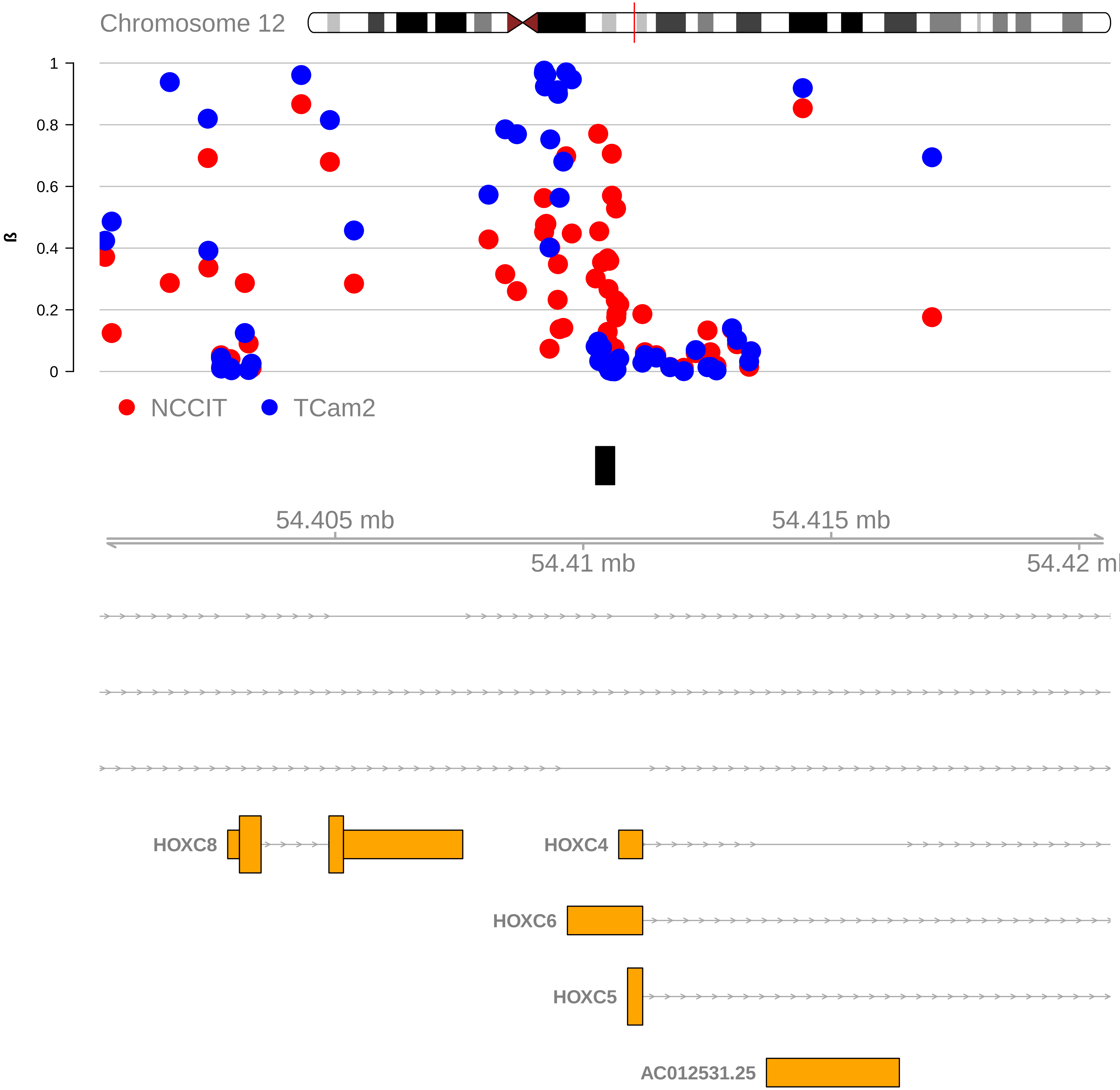
Chromosome 12
ß
1
0.8
0.6
0.4
0.2
0
NCCIT
TCam2
54.405 mb
54.415 mb
54.41 mb
54.42 mb
HOXC8
HOXC4
HOXC6
HOXC5
AC012531.25
